# 

**Published:** 1917-02

**Authors:** 


					﻿PUBLISHED MONTHLY.
SUBSCRIPTION SLOP
ATLANTA
JOLJRNAL-KE;GQR^
OF MEDIClfc7
Atlanta Medical and Surgical Journal, ^steSlisKeJ'.l^SS.	'y
/ and Southern Medical Record, Established 1870.	vJiU I CO I
Vol. LXIII Atlanta, Ga., February, 1917	No. 11
				

